# Synergistic effects of atomically precise Au-based bimetallic nanocluster on energy-related small molecule catalysis

**DOI:** 10.1039/d5sc01108f

**Published:** 2025-04-30

**Authors:** Yuanxin Du, Yi Fang, Pei Wang, Manzhou Zhu

**Affiliations:** a Department of Materials Science and Engineering, Centre for Atomic Engineering of Advanced Materials, Key Laboratory of Structure and Functional Regulation of Hybrid Materials of Ministry of Education, Key Laboratory of Functional Inorganic Material Chemistry of Anhui Province, Anhui University Hefei 230601 China duyuanxin@ahu.edu.cn zmz@ahu.edu.cn

## Abstract

Utilizing renewable sources to convert small-molecule energy carriers (such as nitrogen, carbon dioxide, water, or oxygen) into high value-added chemicals and fuels is of great significance. Rational design of the catalyst is the key to achieving efficient catalytic performance. Atomically precise metal nanoclusters (NCs) exhibit the advantages of high atomic economy, distinctive discrete electronic energy, and homogeneity in size, composition, structure, and surface environment, not only offering extraordinary catalytic activity but also providing the opportunity to reveal the reaction mechanism. In the metal NC family, Au-based NCs have attracted widespread and sustained interest due to their simple preparation, high stability, easy functionalization, and especially their unique catalytic activity, which once provoked a “gold rush” in academia. The synergistic effect between different metal atoms is regarded as an effective strategy to achieve enhanced catalytic performance, but the underlying mechanism is a puzzle. Recently, abundant, diverse and adjustable atomically precise Au-based bimetallic NCs (doped with Ag, Cu, Pt, Pd, Cd, Hg, Ir *etc.*) have emerged, which not only provide a bank of materials for highly active catalysts, but also provide feasibility for revealing synergistic effects at the atomic level. This perspective briefly introduces the common synthesis strategy and structural characteristics of atomically precise Au-based bimetallic NCs, summarizes recent advances in their synergistic catalysis in energy-related small-molecule conversion, and proposes insights and advice for future breakthroughs in this field.

## Abstract 2

This perspective introduces the synthesis strategy and structural characteristics of atomically precise Au-based bimetallic nanoclusters and discusses their synergistic catalysis in energy-related small-molecule conversion.

## Graphical abstract

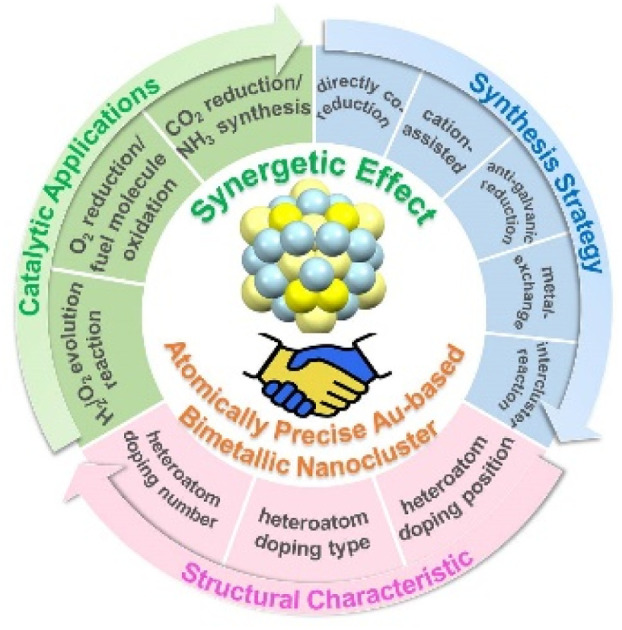

## 1. Introduction

In order to actively tackle the current increasingly severe energy crisis and environmental problems, the search for renewable, clean and environmentally friendly new energy resources has become the consensus and the focus of joint efforts of all countries in the world.^1–4^ Therefore, the catalytic reaction of small molecules related to new energy storage and conversion technologies, such as the hydrogen evolution reaction (HER), the oxygen evolution reaction (OER), the oxygen reduction reaction (ORR), fuel small-molecule oxidation reactions, the carbon dioxide reduction reaction (CO_2_RR), and synthetic ammonia reactions has aroused great interest and attention in academia and industry.^5^ Although some breakthroughs have been achieved after decades of effort and development, these catalytic reactions still face some bottlenecks that need to be overcome. For example, the electrocatalytic water splitting technique for hydrogen production involving HER and OER theoretically requires an applied voltage of 1.23 V in the standard state (25 °C, 1 atm).^6,7^ However, in actual situations, the applied voltage is much higher than the theoretical value. The main reason for this serious deviation from the theoretical voltage phenomenon is that the activity of existing catalysts is not high enough to overcome the reaction energy barrier that OER and HER need to cross as well as solution resistance and contact resistance.^8–10^ For full-cells and metal-air batteries, the critical process is ORR, which not only faces the limitation of sluggish kinetics but also faces the problem of selectivity, because it has two possible pathways: one undergoes a 4-electron transfer step to achieve the complete conversion of O_2_ to H_2_O, and the other is a 2-electron reaction pathway to generate H_2_O_2_.^11–13^ For CO_2_RR, the difficulty in CO_2_ activation due to its chemical inertness and the poor product selectivity due to various possible reduction pathways are stumbling blocks to its further development.^14–17^ The N_2_ reduction reaction (NRR) to synthesize ammonia also faced the N_2_ activation problem. In addition, the diversity of by-product type (*i.e.* H_2_, N_2_H_4_, NO_2_^−^, *etc.*) in NRR or NO_3_RR (NO_3_^−^ reduction reaction) limits the ammonia synthesis efficiency as well.^18–21^ The catalyst is the key to the catalytic reaction, where an excellent catalyst can effectively improve catalytic activity, selectivity, and stability by enhancing the adsorption and activation of the reactant, regulating the adsorption ability of the intermediate, reducing the limiting-rate-step barrier, promoting mass transfer, *etc.*^22–24^ Therefore, accelerating the development of novel highly-efficient catalysts *via* rational design and precise construction is of great significance.

In order to improve the reactivity per unit volume of the catalyst, there are two main strategies: one is to increase the number of active sites and the other is to improve the reaction rate. Catalyst miniaturization is an effective method to realize the former strategy.^25–29^ In recent years, with progress in nanomaterial preparation methods and the rapid development of fine structure characterization techniques, scientists have been able to achieve the controllable preparation of nanoparticles (NPs), from tens of nanometers to precise nanoclusters (NCs) composed of a few to several hundred atoms, and then to single atom catalysts (SACs), maximizing the utilization rate of atoms and achieving atomic economy.^30–32^ The latter is strongly influenced by the adsorption, activation and desorption behaviours of reactants, intermediates and products on the catalyst surface. By using atom doping to exert a synergistic effect between the components, these behaviours can be regulated and optimized to obtain an appropriate and matchable interaction between the reaction molecules and the catalyst, and then realizing an improvement in the catalytic reaction rate.^33–35^ Metal NCs, as an emerging type of nanocatalyst, bridging the gap between metal NPs and SACs, has attracted extensive interest due to the characteristic of having a unique molecule-like discrete electronic energy different from those of metal NPs or SACs.^36–39^ In addition, compared to its large counterpart metal NPs, metal NCs have relatively small size with higher atomic utilization and abundant surface unsaturated coordination active sites.^40–42^ In comparison to SACs, metal NCs exhibit the possibility of a synergistic effect.^43,44^ Furthermore, metal NCs show great adjustability in terms of catalytic properties, because at the sub-nanometer scale, even a single atom change will have a huge impact on the properties of the material.^45–47^

In recent years, research on metal NC catalysis has undergone explosive growth, especially, those metal NCs with precise composition and structure, due to the potential opportunity to investigate the catalytic mechanism based on a clear structure–activity relationship.^32,40,48,49^ Au has for a long time been considered an unreactive noble metal. In the 1980s, Haruta and Hutching *et al.* discovered that small Au NPs exhibit extraordinary catalytic activity in CO oxidation and acetylene hydrochlorination, which has caused a boom in research into Au catalysis.^50,51^ Within the large family of precise NCs, atomically precise Au-based NCs show high stability and ready solubility in various solvents, which is very important for practical catalytic application.^52,53^ In addition, the advantages of easy preparation, high yield, and versatile surface functionalization are also the foundation of Au-based NCs as excellent catalysts.^54–63^ For example, the typical representative, Au_25_ series NCs, can be seen as the standard-bearer of the NC family due to their early discovery (successfully synthesized in 1998 and structurally determined in 2008), and in-depth investigation (properties of optics, chirality, magnetism, electrochemistry, *etc.*), and wide application (catalysis, chemical sensing, imaging, bio-labeling, *etc.*).^64,65^ Furthermore, a variety of derivatives with abundant diversity in terms of properties can be obtained by metal doping, changing ligands, regulating charge states, *etc.*^64–66^

Among various adjustment strategies, heteroatom doping is an effective way to significantly expand the diversity of the composition and structure of NCs and provide a potential synergistic effect for improving catalytic performance. Speaking of a synergistic effect, it is a widely known concept among the public. Simply put, in the field of catalysis, a synergistic effect refers to the catalytic performance produced by the combination of multiple components surpassing that of a single component. It is mainly manifested through synergistic effects between metal atoms, between metals and supports, and between metal atoms and ligands. Here, we focus on the synergistic effects between metal atoms, reflected in the interactions between different metal active centers. Taking the ternary catalyst PtPdRh in automobile exhaust treatment as an example, Pt is mainly responsible for catalyzing the oxidation reaction of CO and CH_*x*_, converting them into non-toxic CO_2_ and H_2_O. Pd also participates in this process, mainly playing a role in heat resistance and improving stability. Rh is mainly responsible for catalyzing the reduction reaction of NO_*x*_, converting them into N_2_ and O_2_. The synergistic effect among the three achieves efficient catalytic activity and stability.

More importantly, the selective and purposeful introduction of foreign atoms into a monometallic NC can rationally optimize catalytic performance *via* modulation of electronic structure, additional active sites, and improvement in structural stability.^43,67,68^ Many researches have reported that Au NCs can be partially substituted with Ag, Cu, Pt, Pd, Cd, Hg, or Ir, and the Au-based alloy NCs perform with superior catalytic activity to their parent Au NCs in numerous reactions due to the synergistic effect.^69^ Motivated by the current era of rapid development of NC materials and energy catalysis, herein, recent advances in atomically precise Au-based bimetallic NCs in energy-related small-molecule catalytic conversion are reviewed, including a summary of common synthesis strategies and structural characteristic of Au-based bimetallic NCs, and a discussion of the synergistic effect on catalytic activity, selectivity, and stability in HER, OER, ORR, CO_2_RR, NRR, NO_3_RR, *etc.*

## 2. Synthesis strategy and structural characteristic of Au-based bimetallic NCs

This section briefly introduces the general synthetic method for Au-based bimetallic NCs and their structural characteristics, such as heteroatom doping number, type and position. The precise and controllable synthesis of materials with high purity and yield is the basis for a benign industrial catalyst. However, synthesizing clusters with precise structures is not an easy task. After extensive attempts and efforts by researchers, it was not until Zhu *et al.* determined the crystal structure of Au_25_(SR)_18_ clusters using X-ray single crystal diffraction in 2008 that the synthesis of atomically precise metal clusters entered a stage of explosive growth.^70^ Our previous review summarized several synthesis strategies for atomically precise metal NCs, such as the direct synthesis method, size-focusing method, ligand-exchange method, chemical etching method, reduction method, metal-exchange method, separation method, intercluster reaction method, and anion-template-assisted method.^48^ In addition, a lot of cluster-related reviews have summarized synthesis methods.^36–39,71^ Besides those mentioned above, others such as mass-selected gas-phase method, template methods, photoreduction method, sonochemical method, microemulsion method, radiolytic method, electrochemical method, and the microwave-assisted method, have also been successfully applied in cluster synthesis. However, not all methods are suitable for the synthesis of bimetallic NCs. Here, we mainly introduce synthesis methods applicable to bimetallic clusters, where common and general methods include the direct co-reduction strategy, cation-assisted strategy, anti-galvanic reduction (AGR) strategy, metal-exchange strategy, and intercluster reaction strategy. The basic principles of these methods are refined and summarized, and corresponding application examples are provided for readers to understand.

Direct co-reduction strategy: It usually takes a metal–ligand complex (*i.e.* Au–SR, Ag–SR, *etc.*) as a precursor and directly synthesizes bimetallic NCs with a reducing agent (*i.e.* NaBH_4_, CO, *etc.*); it is also called “*in situ*” or “one-pot” synthesis.^48,69^ For example, Au_25−*x*_Ag_*x*_(SR)_18_ NCs with continuously modulated *x* have been successfully synthesized by this method.^72^

Cation-assisted strategy: this refers to the synthesis of bimetallic NCs by the reaction of the existing parent NCs with another metal cation.^69^ For instance, Murray *et al.* obtained Au_25−*x*_Ag_*x*_(SR)_18_ NCs by utilizing Ag^+^ ions reacting with Au_25_(SR)_18_ NCs.^73^

Anti-galvanic reduction (AGR) strategy: Wu *et al.* proposed that the redox potential of the order of metals is no longer the only decisive thermodynamic parameter when the size of NCs is less than 3 nm.^74^ They prepared Au–Ag and Au–Cu bimetallic NCs by doping heteroatoms in Au_25_(SR)_18_ on the basis of the AGR method.^75,76^

Metal-exchange strategy: instead of using an inorganic metal salt, an organic metal–ligand complex is used in the synthesis of bimetallic NCs, and this synthesis process can no longer relate to the rule of metal redox potential.^77^ For example, our group utilized Au_25_(SR)_18_ as a template to synthesize Cd_1_Au_24_(SR)_18_ and Hg_1_Au_24_(SR)_18_ by this method.^78^

Intercluster reaction strategy: this refers to the preparation of new bimetallic NCs *via* the reaction between two stable NCs.^48,69^ For example, (Au_25−*x*_Ag_*x*_)(SR)_18_ and Au_*x*_Ag_44−*x*_(SR)_30_ NCs are obtained by spontaneously exchanging metal atoms after mixing Au_25_(SR)_18_ and Ag_44_(SR)_30_.^79^

**Fig. 1:**
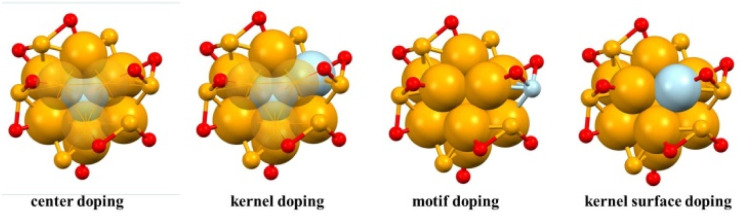
Schematic diagram of the doping position of the heteroatom in Au-based bimetallic NCs. Atom colors: Au = orange, heteroatom = blue, S = red.

For bimetallic NCs formed by heteroatom doping, the most important considerations are the type, number and position of the doping atoms, because these structural parameters have a great influence on the catalytic performance. Considering that NCs can be viewed as being composed of a metal kernel and a metal–ligand motif, the doping position of the heteroatom can be divided into center doping, kernel doping, kernel surface doping, and motif doping (Fig. 1). For motif doping, the heteroatom is connected with the coordination atom of the ligand, such as S, P, or N atoms. It is located on the outside of the cluster and has the opportunity to make direct contact with the reactant. For kernel doping (including center doping and kernel surface doping simultaneously) and kernel surface doping, the heteroatom is located at the surface of the metal core. When the catalytic reaction occurs, some ligands will detach due to the weak connection between metal atoms and ligands, and the atoms at the surface of the metal core are exposed as active centers to directly participate in the reaction. For center doping, the heteroatom is located at the center of the metal core, and it seems to have no influence on catalysis due to the indirect contact with the reactant. However, it can participate in the catalytic reaction by modulating the electronic structure of the metal core, regulating the interaction strength with the reactant or intermediate, and optimizing the reaction pathway and kinetics. The synthesis methods and characteristic structural parameters of Au-based bimetallic NCs reported in recent years are summarized in Appendix Table 1.

## 3. Au-based bimetallic NCs in energy-related catalysis

Aiming at current attractive energy-related catalytic reactions, this part classifies, summarizes, and discusses recent progress in the synergistic effects of Au-based bimetallic NCs in the hydrogen evolution reaction, oxygen evolution reaction, oxygen reduction reaction, fuel small-molecule oxidation reaction, carbon dioxide reduction reaction, and synthetic ammonia reaction.

### 3.1 Au-based bimetallic NCs in the hydrogen evolution reaction

The gradual depletion of fossil fuels and consequent environmental and climate concerns have stimulated the development and use of clean and renewable energy sources. Among various clean energy carriers, hydrogen with its high energy density is considered to be one of the promising energy resources for a sustainable society.^80^ The hydrogen evolution reaction (HER), which uses the abundant water on Earth as a raw material and is driven by renewable energy, such as hydropower, photovoltaic, wind, or tidal energy, is an ideal method of hydrogen production, which has many advantages, including environmental friendliness, production of high-purity hydrogen, and low carbon emissions.^81^ Currently, the mainstream prediction method for the superiority of the HER catalyst is based on the volcano plot, which is constructed by linking the hydrogen adsorption energy with the exchange current.^82^ According to the Sabatier principle, a good HER catalyst does not bind the hydrogen too weakly or too strongly; therefore, a nearly thermodynamically neutral hydrogen binding step is assurance of a good HER catalyst.^83^

**Fig. 2:**
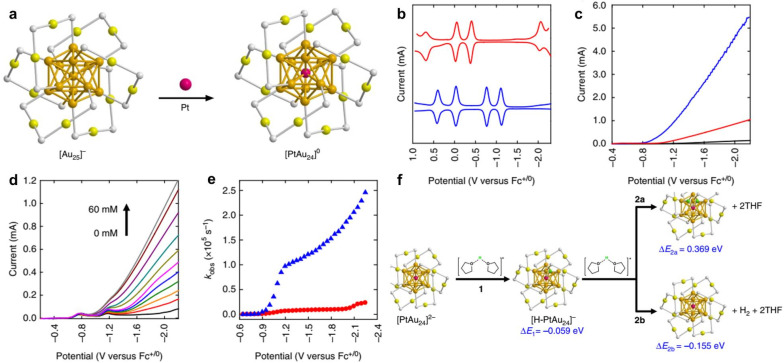
(a) Structure of Au_25_ and Pt_1_Au_24_ NC. (b) SWVs of Au_25_ (red) and Pt_1_Au_24_ (blue) in CH_2_Cl_2_ (0.1 M Bu_4_NPF_6_). (c) LSVs in THF (0.1 M Bu_4_NPF_6_) solution containing 1.0 M TFA without a catalyst (black) and with Au_25_ (red) and Pt_1_Au_24_ (blue). (d) LSVs of Pt_1_Au_24_ in THF (0.1 M Bu_4_NPF_6_) with different concentrations of TFA. (e) *K*_obs_–potential plots for Au_25_ (red) and Pt_1_Au_24_ (blue). (f) Reaction energy calculation for HER on Pt_1_Au_24_. Reproduced from ref. 87 with permission from Springer Nature, copyright 2017.

At present, Pt is recognized as the best-known efficient HER electrocatalyst; however, its high cost and scarce stocks hamper its large-scale industrial application.^84^ As the sixth period element in the periodic table next to Pt, the electronic structure of Au is similar to that of Pt, so there is reason to believe that Au NCs have the potential to be an outstanding HER catalyst.^85,86^ Kwak *et al.* selectively replaced the center Au atom in Au_25_ NCs with a single Pt atom and obtained Pt_1_Au_24_ bimetallic NCs (Fig. 2a).^87^ Pt doping has little effect on the geometric structure of the NCs; Pt_1_Au_24_ almost maintains the configuration of the original Au_25_. However, the electronic structure changes a lot, which is reflected in the surface charge state ([Pt_1_Au_24_]^0^ & [Au_25_]^−^), superatomic electronic configuration (Au_25_: 8-electron, Pt_1_Au_24_: 6-electron)^88–90^ and optical absorbance spectra (Au_25_ : 1.8 eV, Pt_1_Au_24_ : 1.1 and 2.1 eV). The redox behavior is also drastically altered. As measured by square-wave voltammetry (SWV), the gaps between the first oxidation (O1) and reduction (R1) potential for Au_25_ and Pt_1_Au_24_ are 1.67 and 0.73 V, respectively (Fig. 2b). The HOMO–LUMO (highest occupied-lowest unoccupied molecular orbitals) gaps are 1.32 and 0.29 V for Au_25_ and Pt_1_Au_24_, respectively. It is worth noting that the reduction potential of Pt_1_Au_24_ is nearly 1 V more positive than that of Au_25_, indicating the possibility of a lower overpotential for the electrocatalytic reduction reaction. This conjecture is further confirmed by the result of linear sweep voltammograms (LSVs). In THF (0.1 M Bu_4_NPF_6_) solution containing 1.0 M trifluoroacetic acid (TFA), Au_25_ and Pt_1_Au_24_ show onset potentials at −1.10 and −0.89 V, respectively (Fig. 2c). Notably, the overpotential for Pt_1_Au_24_ is ∼70 mV (relative to −0.82 V, the thermodynamic reduction potential of a proton in THF with 1.0 M TFA), which is superior to other natural hydrogenase enzymes (∼100 mV).^91–93^ With an increase in TFA concentration, the current at −0.76 V (the first reduction [Pt_1_Au_24_]^0^/^−^) of Pt_1_Au_24_ shows no significant change, while the current at −1.10 V (the second reduction [Pt_1_Au_24_]^−^/^2−^) is significantly enhanced, suggesting that Pt_1_Au_24_ acts as an electron transfer mediator for HER (Fig. 2d).^94,95^ The *k*_obs_ (pseudo-first-order rate constant) of Pt_1_Au_24_ is calculated to be 121000 s^−1^ at *η* = 650 mV, which is higher than that of Au_25_ (8000 s^−1^) or other molecule-like complexes (Co-complex: 700 s^−1^ at *η* = 890 mV, Cu-complex: 11000 s^−1^ at *η* = 720 mV, Ni-complex: 106000 s^−1^ at *η* = 650 mV) (Fig. 2e).^96–98^

In addition, Pt_1_Au_24_ shows charge-state-dependent catalytic activity, indicating molecule-like catalytic behavior different from that of the larger-sized metal NPs. The currents at potentials negative to the [Pt_1_Au_24_]^−/2−^ all exhibit linear correlation with [Pt_1_Au_24_] and [TFA]^1/2^, suggesting a heterolytic HER mechanism, [H–Pt_1_Au_24_]^−^ + H^+^ → [Pt_1_Au_24_]^0^ + H_2_. In contrast, the current at the potential at which [Pt_1_Au_24_]^−^ exists as the predominant form shows linear correlation with [TFA] and [Pt_1_Au_24_]^3/2^, indicating a homolytic HER mechanism, [H–Pt_1_Au_24_]^0^ + [H–Pt_1_Au_24_]^0^ → 2[Pt_1_Au_24_]^0^ + H_2_. The heterolytic HER mechanism over Pt_1_Au_24_ is further confirmed by density functional theory (DFT) calculations with an energy change of −0.155 eV, while for Au_25_, the thermodynamically favorable HER pathway is the homolytic mechanism (Fig. 2f). Additionally, an H–Pt bond can spontaneously form. The bond length between adsorbed H and central Pt is shorter than that between adsorbed H and surface Au, indicating that the stronger H–Pt interaction is beneficial for HER energetics on [Pt_1_Au_24_]^2−^. As a result, Pt_1_Au_24_ exhibits excellent HER activity whether for homogeneous catalysis in non-aqueous solvent or heterogeneous catalysis in aqueous media, compared to Au_25_, other molecule-like complexes, or commercial Pt/C benchmarking.

**Fig. 3:**
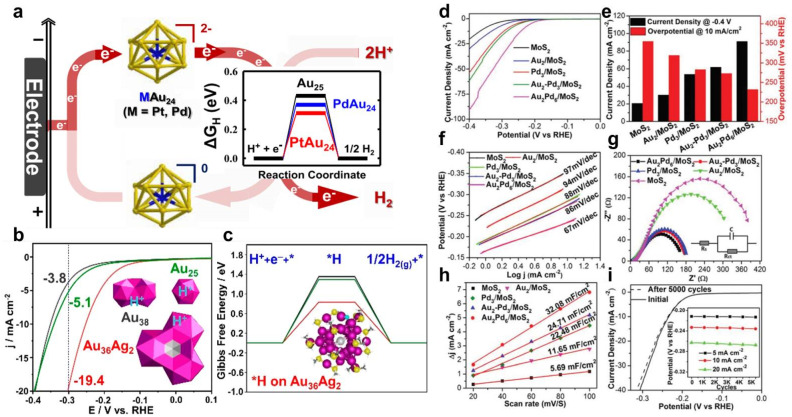
(a) Schematic diagram of M_1_Au_24_ (M = Pt, Pd) enhanced HER activity compared to Au_25_ NCs. Reproduced from ref. 100 with permission from American Chemical Society, copyright 2018. (b) LSVs and (c) Δ*G*_H_ of Au_25_, Au_38_, and Au_36_Ag_2_. Reproduced from ref. 102 with permission from American Chemical Society, copyright 2021. (d) LSVs, (e) current density and overpotential comparison, (f) Tafel slope, (g) electrochemical impedance spectroscopy, and (h) electrochemical double-layer capacitance of MoS_2_, Au_2_/MoS_2_, Pd_3_/MoS_2_, Au_2_–Pd_3_/MoS_2_, and Au_2_Pd_6_/MoS_2_. (i) Stability test of Au_2_Pd_6_/MoS_2_. Reproduced from ref. 103 with permission from Royal Society of Chemistry, copyright 2018.

Recently, Sun *et al.* revisited the electrocatalytic HER activity origin of Pt_1_Au_24_ NCs by a combination of advanced first-principles calculations and attenuated total reflection surface-enhanced infrared absorption spectroscopy (ATR-SEIRAS) experiments.^99^ They found that in addition to the central Pt atom, the exposed bridged Au site, due to spontaneous thiolate ligand desorption during the electrochemical process, is also a catalytically active site. The synergistic effect between the two types of active site contributes to the extraordinary HER activity of the Pt_1_Au_24_ cluster. As Pt centrally doped Pt_1_Au_24_ significantly improves HER activity, it is natural for researchers to ask whether Pd_1_Au_24_, which has a nearly isoelectronic structure and similar redox property, would also exhibit similarly high HER activity. Choi *et al.* systematically compared the HER catalytic activity parameters, such as onset potential (*E*_onset_), current density, and turnover frequency (TOF) of M_1_Au_24_ and M_2_Au_36_ (M = Pt, Pd).^100^ Compared to non-doped Au NCs, atom doping not only brings about a change in the electrochemical redox property, but also causes an improvement in HER activity. *E*_onset_ is determined by the match between the reduction potential of NC and H^+^. In both M_1_Au_24_ and M_2_Au_36_ systems, Pt doping shows higher current density and TOF than Pd doping, which is attributed to the lower H adsorption free energy (Δ*G*_H_) in Pt-doped Au NCs (Fig. 3a). Based on the excellent HER performance of Pt_1_Au_24_, Negishi *et al.* further changed the surface ligand to, for example, C6 = 1-hexanethiolates, TBBT = 4-*tert*-butylbenzenethiolate, PDT = 1,3-propanedithiolate, or PET = 2-phenylethanethiolate, in pursuit of higher catalytic activity.^101^ Due to the differences in the lengths and orientations of -Au(i)-SR-Au(i)- staples between TDT/PDT- and C6/PET-protected NCs, the Au atoms of the Pt_1_Au_12_ metal core in TDT/PDT-protected Pt_1_Au_24_ NCs are exposed on the outside, resulting in enhanced HER activity. Although Ag is considered to be inactive for HER, obtaining AuAg alloy NCs with comparable HER activity is highly possible *via* a reasonable design. Li *et al.* synthesized Au_36_Ag_2_(SR)_18_ NCs, which can be regarded as having a trimeric structure, that is with three icosahedral (*I*_h_) units face-fused together in a cyclic manner.^102^ The unique face-fusion mode in Au_36_Ag_2_ endows it with unfilled superatomic orbitals, a low ligand-to-metal ratio, and low-coordinated Au atoms, leading to lower Δ*G*_H_ and higher electron affinity, thus showing improved HER activity over its counterpart monomeric Au_25_ and dimeric Au_38_ (Fig. 3b and c).

In addition to the synergies between different metal components within clusters, there are also interactions between NCs and functional carriers, which can be combined in one system to maximize synergies. Du *et al.* designed and synthesized Au_2_Pd_6_ NCs, which can be considered to be two Pd_3_ NCs connected by an Au_2_ unit.^103^ On the one hand, after MoS_2_ couple metal NCs, the number of catalytic active sites is increased, and electron transport is promoted, resulting in enhanced HER activity. On the other hand, compared to monometallic Au_2_ and Pd_3_ NCs, and mixed Au_2_–Pd_3_ NCs, bimetallic Au_2_Pd_6_ shows the best HER activity with the lowest overpotential, the smallest Tafel slope and electrochemical resistance, and the largest electrochemically active surface area and TOF value (Fig. 3d–i). X-ray photoelectron spectroscopy and Raman results indicate the strong electronic interaction between NCs and MoS_2_. DFT calculations demonstrate that the largest number of catalytic sites with almost nearly zero Δ*G*_H_ occur on Au_2_Pd_6_/MoS_2_, further explaining the excellent HER activity of Au_2_Pd_6_/MoS_2_ from a theoretical perspective. A similar phenomenon is observed on bimetallic Au_4_Cu_2_ NCs, which show better HER activity than that of monometallic Cu_6_ or Au_6_ NCs.^104^ Taking atomically precise alloy NCs as a precursor to proceed with ligand-assisted pyrolysis is a novel method to form nanocluster/single atom (NC/SA) composite systems. Lv *et al.* obtained a hybrid system containing two different active sites types, AuPd alloy NCs and satellite Pd SAs (AuPd_NCs_/Pd_SAs_), by controllable thermal treatment of Au_4_Pd_2_(SC_2_H_4_Ph)_8_.^105^ In the hybrid system, the satellite Pd SAs play the role of optimizing the electronic structure, and the Au sites effectively promote the adsorption and dissociation of H_2_O molecules. Thereby, the synergy between Au and Pd promotes the excellent HER activity of AuPd_NCs_/Pd_SAs_-600, which shows one order of magnitude higher mass activity and TOF than commercial Pd/C or Pt/C.

**Fig. 4:**
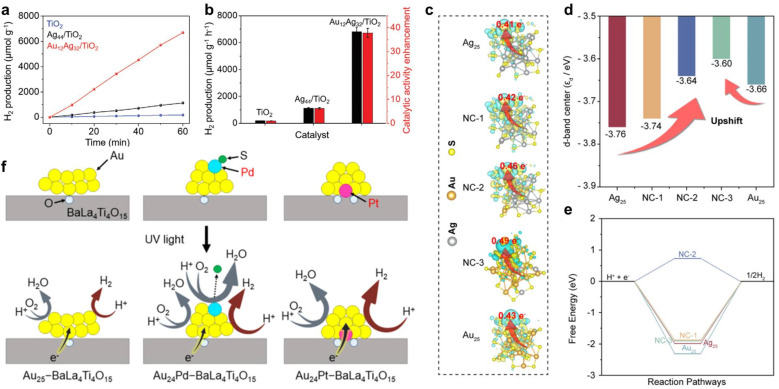
(a and b) Comparison of photocatalytic HER activity of TiO_2_, Ag_44_/TiO_2_, and Au_12_Ag_32_/TiO_2_. Reproduced from ref. 107 with permission from Wiley-VCH, copyright 2023. (c) The isosurfaces of charge density difference of H*-adsorbed NCs. (d) D-band center and (e) Δ*G*_H_ of [Au_*x*_Ag_25−*x*_(SR)_18_]^−^ series NCs. Reproduced from ref. 108 with permission from Royal Society of Chemistry, copyright 2023. (f) Proposed structures of M_1_Au_24_–BaLa_4_Ti_4_O_15_ for M = (Au, Pd, Pt) before (top) and during (bottom) the water splitting reaction. Reproduced from ref. 109 with permission from American Chemical Society, copyright 2019.

In addition to electrocatalytic HER, solar-driven photocatalytic water splitting is a sustainable and clean approach to produce hydrogen. Metal NCs exhibit a molecule-like discrete electronic energy band and strong light absorption in broad spectra, which meet the basic requirements for potential promising photocatalysts.^106^ Bootharaju *et al.* synthesized a selenolated [Au_12_Ag_32_(SePh)_30_]^4−^ core–shell cluster based on a templated galvanic exchange strategy, which is comprised of an Au icosahedral core and an Ag_12_(SePh)_30_ shell.^107^ The synergistic effect between the Au core and the Ag shell greatly changes the overall electronic structure, which aligns well with the band structure of TiO_2_, facilitating photogenerated charge carrier separation. Therefore, Au_12_Ag_32_/TiO_2_ shows a photocatalytic H_2_ production rate of 6810 μmol g^−1^ h^−1^ under solar irradiation, which is 37.8 and 6 times higher than those of TiO_2_ and Ag_44_/TiO_2_, respectively (Fig. 4a and b). Liu *et al.* obtained [Au_*x*_Ag_25−*x*_(SR)_18_]^−^ series NCs (1 ≤ *x* ≤ 3, 3 ≤ *x* ≤ 8, 19 ≤ *x* ≤ 23, defined as NC-1, NC-2, NC-3).^108^ Under visible-light irradiation, the H_2_ production rate follows the order: NC-3 > NC-2 > NC-1 > Au_25_ > Ag_25_. As revealed by DFT calculation, bimetallic Au_*x*_Ag_25−*x*_ shows that the better photocatalytic HER activity is due to the optimization of electronic structure caused by the Au–Ag synergy. The narrowed HOMO–LUMO gap in Au_*x*_Ag_25−*x*_ partially contributes to the better HER performance. What is more, the microenvironment localized in dual Au–Ag sites benefits the formation of electron-acceptor centers, resulting in photogenerated electrons tending to remain in a cluster to reduce the adsorbed H^+^ (Fig. 4c). Additionally, the Au–Ag bimetallic synergistic effect upshifts the D-band centers and balances hydrogen adsorption/desorption dynamics (Fig. 4d and e). Kurashige *et al.* loaded Pd_1_Au_24_ and Pt_1_Au_24_ on BaLa_4_Ti_4_O_15_*via* stepwise ligand-exchange, adsorption, and calcination processes, to investigate the effect of heteroatom doping type on photocatalytic HER activity.^109^ Although the Pd and Pt atoms are both in the center of M_1_Au_24_ when it exists in the form of NCs alone, the positions of Pd and Pt atom are different after depositing them on BaLa_4_Ti_4_O_15_. As can be rationally inferred from extended X-ray absorption fine structure (EXAFS) spectroscopy results, Pd is located at the surface of the NCs, while Pt is at the interface between NCs and BaLa_4_Ti_4_O_15_. Whether Pd or Pt doping is used, the electron density of Au in the NCs is improved. However, for photocatalytic HER activity opposite results are obtained, where Pt doping increases the activity; in contrast, Pd doping causes a decrease in activity, which to a great degree can be attributed to the doping position of the heteroatom (Fig. 4f).

### 3.2 Au-based bimetallic NCs in the oxygen evolution reaction

**Fig. 5:**
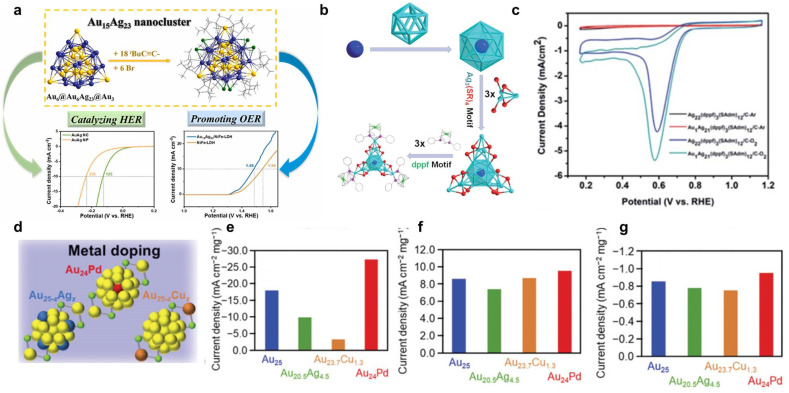
(a) Schematic diagram of Au_15_Ag_23_ NC as efficient HER and OER catalyst. Reproduced from ref. 112 with permission from Elsevier, copyright 2024. (b) Structural analysis of Au_1_Ag_21_ NC. (c) Comparison of ORR performance of Au_1_Ag_21_ and Ag_22_ NC. Reproduced from ref. 124 with permission from Royal Society of Chemistry, copyright 2021. (d) Structural scheme of metal-doped Au_25_ NC. Comparison of (e) HER, (f) OER, and (g) ORR activity of Au_25_ and different metal-doped Au_25_ NCs. Reproduced from ref. 127 with permission from Royal Society of Chemistry, copyright 2020.

The oxygen evolution reaction (OER) is an elementary reaction of great importance in the field of energy and the environment. It refers to the half-reaction that occurs on the anode during electrolysis of H_2_O. Because OER involves a four-electron transfer process, it exhibits slow reaction kinetics, and requires higher energy to trigger a reaction; therefore, it is the key factor restricting the efficiency of an overall H_2_O electrolysis device.^110,111^ Liu *et al.* synthesized an alkynyl-protected AuAg alloy cluster, Au_15_Ag_23_(^*t*^BuC≡C)_18_Br_6_, which has a triple-layered core–shell–shell (Au_6_@Au_6_Ag_23_@Au_3_) configuration.^112^ Au_15_Ag_23_ not only shows excellent electrocatalytic HER activity (*η* = 125 mV to reach 10 mA cm^−2^ current density) but it also displays an outstanding OER property when it is loaded on NiFe layered double hydroxide, whose overpotential is 250 mV to reach 10 mA cm^−2^ (Fig. 5a). In an overall water splitting (OWS) system, achieving 10 mA cm^−2^ requires only 1.51 V, and it performs for as long as 50 h in a stability test.

The electrocatalytic OWS performance of Au NCs can be regulated by utilizing the electron-attracting ability of an Fe atom to Au NCs. Sun *et al.* synthesized a hybrid system of Au–Fe_1_NCs loaded on Ni foam to act as alkaline HER and OER bifunctional electrocatalysts.^113^ The electron structure of H adsorption on the Au NC surface is optimized by the introduction of Fe atoms, manifested by a low overpotential of 35.6 mV to reach 10 mA cm^−2^ current density in HER. In addition, the hybrid system Au–Fe_1_NCs display good OER activity with a 246 mV low overpotential at 10 mA cm^−2^ oxidation current density. In a two-electrode alkaline OWS device, the cell voltage to reach 10 mA cm^−2^ current density is only 1.52 V, and the durability test lasts for 40 h. The synergistic effect in Au–Fe_1_NCs is elucidated by X-ray photoelectron spectroscopy (XPS) experiments and DFT theoretical calculations, mainly reflected in the promotion of electron delocalization and enhancement of O–H bond activation of Au NCs and an upshift in the D-band center of the Fe atom.

The synergistic effect induced by heteroatom doping has shown great improvement in electrochemical activity; however, the understanding of the effect on photo-/photoelectro-catalytic activity, especially that involving charge transfer characteristics, remains blank. Su *et al.* took glutathione-protected Ag-doped Au NCs (Au_1−*x*_Ag_*x*_@GSH) and a non-conjugated insulating polymer of poly(diallyl-dimethylammonium chloride) (PDDA), as negatively and positively charged building blocks to elaborately fabricate a spatially multilayered alloy NCs/metal oxide photoanode heterostructure *via* layer-by-layer (LBL) assembly.^114^ Au_1−*x*_Ag_*x*_@GSH acts as a photosensitizer that can be instantly photoexcited to generate e^−^ and h^+^, and PDDA plays the role of an electron-withdrawing mediator to accelerate interfacial charge transfer and form a cascade electron transport channel. As a result, LBL-assembled TiO_2_/(PDDA-Au_1−*x*_Ag_*x*_)_*n*_ shows good photoelectrochemical H_2_O oxidation performance under visible-light irradiation.

### 3.3 Au-based bimetallic NCs in the oxygen reduction reaction

The electrocatalytic oxygen reduction reaction (ORR) is the process by which oxygen is reduced to water or other oxygen-containing species in an electrochemical system. ORR usually has two reaction pathways, one of which is the 4-electron reduction process, starting from O_2_ to H_2_O, and it is one of the key reactions in energy conversion devices such as fuel cells and metal-air batteries, and its efficiency has a decisive impact on the performance of these devices.^115,116^ While the other one is the 2-electron pathway, converting O_2_ to H_2_O_2_, which can serve as a promising alternative to the traditional energy-intensive anthraquinone process.^117–119^

According to theoretical calculation, the decrease in the core size of Au NPs will result in a narrowed D-band and shift to the Fermi level, which are considered to be beneficial for O_2_ adsorption.^120,121^ At an early stage, Chen *et al.* utilized two series of Au NCs with different core sizes to investigate the effect of size on ORR activity, and they found the ORR catalytic activity of both follows the trend where a smaller size of NCs results in better ORR reactivity (Au_11_ > Au_25_ > Au_55_ > Au_140_ and Au_25_ > Au_38_ > Au_144_).^122,123^ Besides the core size of the NCs, heteroatom doping is another critical factor influencing the ORR activity of Au NCs. Zou *et al.* synthesized M_1_Ag_21_(dppf)_3_(SAdm)_12_](BPh_4_)_2_ (M = Au/Ag) by introducing 1,1′-bis-(diphenylphosphino)-ferrocene (dppf) as an activating ligand.^124^ Dppf is a derivative of ferrocene as well as an electron donor, acting as a common protective ligand used to design and synthesize a metal complex with excellent electrochemical activity. Compared to other Au/Ag NCs without a dppf ligand, the two NCs show better ORR activity. In addition, the two NCs exhibit the same icosahedral M_13_ (M = Au/Ag) kernel with a protective shell of 3 Ag_3_(SR)_4_ motifs and the protection of 3 dppf ligands (Fig. 5b). The identical geometric structure provides an ideal platform to reveal the heteroatom doping effect. Au_1_Ag_21_ shows higher onset potential and diffusion limited current density, indicating better ORR activity than that of Ag_22_ (Fig. 5c).

Xu *et al.* simultaneously introduced a dppf surface ligand and a Cd surface doping atom to perform a surface engineering strategy, and then obtained Au_27_Cd_1_(SAdm)_14_(dppf)Cl NCs.^125^ Au_27_Cd_1_ and Au_38_ NCs have a similar bi-icosahedral Au_23_ core, but different surface structures due to surface engineering. The reconstructed surface structure offers enhanced ORR activity for Au_27_Cd_1_. The synergistic effect between Au and Cd is not only reflected in a comparison of the ORR catalytic activity of Au_27_Cd_1_ and Au_38_ NCs but is also manifested in the improved ORR activity of Au_22_Cd_1_ compared to that of Au_24_ NCs. Furthermore, based on the Koutecky–Levich plots, the overall number of electrons transferred for Cd-doped Au NCs is higher than that for bare Au NCs (Au_38_ : 2, Au_27_Cd_1_: 3.3, Au_24_ : 2.2, Au_22_Cd_1_: 3), indicating that Cd doping will change the ORR reaction pathway of Au NCs. In addition to Ag/Cd doping, AuPd alloy NCs show better ORR activity than Au NCs. Yan *et al.* synthesized GSH-protected AuPd NCs and tuned the structure and composition with the Pd-to-Au ratio.^126^ The AuPd NCs are supported on carbon nanosheets and the protective ligand is completely removed by calcination. The hybrid composite with 30% metal mass loading and Pd-to-Au ratio of 1 : 2 exhibits optimized ORR activity.

Although there is lots of research into Au_*n*_(SR)_*m*_-related alloy NCs in electrocatalytic HER, OER, and ORR, the activities are obtained under different experimental conditions, so it is difficult to make a deep comparison and develop a general rule about the methods needed to achieve high activity. Kumar *et al.* systematically investigated the effect of heteroatom species on the activity of these reactions.^127^ Compared to Cu/Ag doping, Pd-doped Au NCs show the highest current density in all three reactions (Fig. 5d–g). Other parameters, such as the number of constituent atoms or ligand functional groups, are also carefully evaluated. The results indicate that the decreased number of constituent atoms and thickness of the ligand layer are helpful for an improvement in activity in all three reactions.

### 3.4 Au-based bimetallic NCs in the fuel small-molecule oxidation reaction

A fuel cell is a chemical device that converts the chemical energy of a fuel directly into electrical energy. Theoretically, it can operate at close to 100% thermal efficiency. It exhibits lots of advantages, such as high economy, low environmental pollution, high reliability, a wide selection of fuels and easy construction.^128–132^ Depending on the type of fuel entering at the anode, different types of fuel cells can be obtained. For example, a direct ethanol fuel cell (DEFC) is a device that uses ethanol with its merits of high energy density, low cost and easy storage as a fuel to perform an anodic oxidation reaction and convert it into electric energy.^133–135^ Au NCs exhibit excellent performance in the ethanol oxidation reaction (EOR). Tang *et al.* reported alkynyl-protected Au_28_(DMPA)_20_ NCs (DMPA = 1-ethynyl-2,4-dimethylbenzene) synthesized by a direct reduction method.^136^ Compared to thiol-ligand-protected Au_28_(SR)_20_ (here SR = TBBT/CPT, TBBT = 4-tertbutylbenzenethiol, CPT = cyclopentanethiol) with a similar structure, Au_28_(DMPA)_20_ shows superior EOR mass activity and specific activity. In order to further improve the EOR activity, commercial Pd and Pt are introduced into Au NCs as heteroatom dopants. Zhang *et al.* synthesized surface-clean Pt_3_Au NCs with a uniform size of 2.1 nm supported on PDDA-functionalized graphene (Pt_3_Au@PDDA-G) by a CO reduction method.^137^ The obtained Pt_3_Au NCs not only effectively suppress Ostwald ripening but also show enhanced EOR activity due to the more efficient removal of the adsorbed CO-like intermediates based on the synergistic effect between Pt and Au. Similarly, Cui *et al.* utilized a wet-chemical method to synthesize AuPd NCs with an average size of 1.0 nm supported on amine-functionalized carbon black (AuPd/CB_H−A_).^138^ In EOR, Au_0.4_Pd_0.6_/CB_H−A_ exhibits 5.25 A mg_AuPd_^−1^ mass activity and 5.98 mA cm_AuPd_^−2^ specific activity, which are 9.7 and 3.4 times greater than those of commercial Pd/C (0.54 A mg_Pd_^−1^ and 1.74 mA cm_Pd_^−2^), respectively.

In addition, direct formic acid fuel cells (DFAFCs) fueled by formic acid are considered to be one of the most promising power sources for portable electronic devices in the future due to their small size, low toxicity and low penetration of Nafion membranes.^139,140^ Pt is generally considered to be the most effective catalyst towards the anodic reaction of DFAFCs, formic acid oxidation (FAO). However, due to its poor resistance to CO toxicity, its current development faces a bottleneck. Lu *et al.* synthesized single-Pt-atom-doped Au_25_ (Pt_1_Au_24_(SR)_18_) NCs.^141^ Although only one Pt replaces the central Au atom, Pt_1_Au_24_ shows excellent FAO performance with a high mass activity of 3.7 A mg_Pt+Au_^−1^, which is 12 and 34 times higher than that of Pt NCs and commercial Pt/C, respectively. Moreover, the unavailability of adjacent Pt atoms in Pt_1_Au_24_ NCs effectively suppresses the poisoning of CO intermediates, which is reflected in the outstanding performance in an accelerated durability test and CO stripping experiment. On the basis of *in situ* electrochemical FTIR observation and thermodynamic and kinetic calculation, the FAO process on Pt_1_Au_24_ is speculated to be a direct pathway with COOH* as the preferred reactive intermediate. The introduction of a Pt atom leads to a decreased HOMO–LUMO gap and higher reactivity, and the original Au atomic shell plays a CO anti-poisoning role. The synergistic effect in Pt_1_Au_24_ NCs contributes to the high activity and good stability.

### 3.5 Au-based bimetallic NCs in the carbon dioxide reduction reaction

The catalytic conversion of carbon dioxide is the process of converting CO_2_ into chemicals, energy products and functional materials to achieve resource utilization, which has great significance and application prospects for sustainable development. The use of low-grade renewable electricity to reduce CO_2_, which can both reduce CO_2_ emissions and “turn waste into good”, and convert renewable energy into high energy density fuel storage, is considered a green and promising technique due to its flexible and sustainable method of conversion.^142–146^

**Fig. 6:**
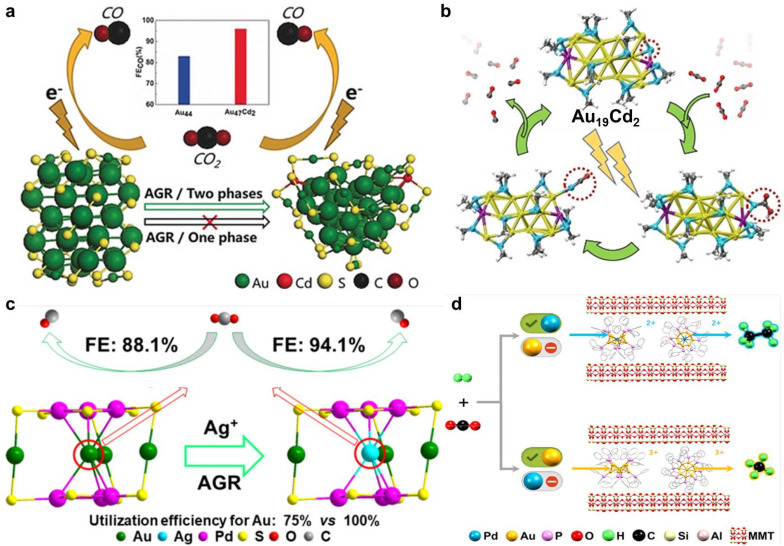
(a) Schematic diagram of the synthesis and comparison of the CO_2_RR activity of Au_44_ and Au_47_Cd_2_ NCs. Reproduced from ref. 148 with permission from Wiley-VCH, copyright 2020. (b) Schematic diagram of the structural dynamic evolution of Au_19_Cd_2_ during CO_2_RR. Reproduced from ref. 149 with permission from Wiley-VCH, copyright 2021. (c) Synthesis scheme and comparison of the CO_2_RR activity of Au_4_Pd_6_ and Au_3_AgPd_6_ NCs. Reproduced from ref. 152 with permission from American Chemical Society, copyright 2022. (d) Schematic diagram of tuning selectivity in CO_2_ catalytic conversion by Au_9_ and Au_8_Pd_1_ NCs. Reproduced from ref. 153 with permission from Chinese Chemical Society, copyright 2021.

At present, the possible main products of CO_2_ electroreduction include C_1_ products (*e.g.*, carbon monoxide, formic acid, methane, methanol) and C_2+_ chemicals (*e.g.*, ethylene, ethanol, ethane, *n*-propanol).^145^ Among the different products of CO_2_RR, the conversion of CO_2_ to CO is considered to be one of the most promising reactions in the chemical industry due to its technical and economic feasibility.^147^ Due to the relatively weak binding of *CO intermediates to Au, Au-based materials show particularly high selectivity for the formation of CO. It is believed that alloying or doping can further improve the CO_2_RR efficiency of Au NCs by optimizing the electronic structure and surface geometry. Zhuang *et al.* employed Au_44_(TBBT)_28_ as the parent NCs and chose Cd^2+^ as the oxidative ions to prepare Au_47_Cd_2_(TBBT)_31_ NCs by the anti-galvanic reduction (AGR) method in a two-phase system.^148^ The doped Cd atoms are on the surface and are coordinated by three thiolates. Compared to Au_44_(TBBT)_28_, Au_47_Cd_2_(TBBT)_31_ shows higher faradaic efficiency (FE) for CO over a wide potential range from −0.3 V to −0.9 V, displaying FE_CO_ up to 96% at −0.57 V (Fig. 6a). DFT calculations indicate that introducing Cd atoms results in a change in the adsorption configuration for the COOH* intermediate. Unlike Au_44_(TBBT)_28_, the O atom of COOH* prefers to bind with Cd, leading to the formation of Cd–O–C(OH)–Au and decreased COOH* formation energy, benefiting the enhancement in CO_2_RR activity.

In addition, Li *et al.* synthesized Cd-surface-modified Au NCs (Au_19_Cd_2_(SR)_16_, SR = cyclohexanethiolate) by taking Au_23_(SR)_16_ as the template.^149^ Compared to Au_23_, the kernel of Au_19_Cd_2_ is maintained, except that two surface Au atoms are replaced by Cd dopants. The introduction of Cd obviously improves the selectivity and activity of CO formation. The determination of active sites is investigated in detail. Compared to the inactivity of fully ligand-protected NCs, partial ligand detachment is considered a necessity for high catalytic activity. However, it needs to be carefully studied which part (–R or –SR) it is reasonable to remove, and which exposure site (S or Au) is the real catalytic site. Through DFT calculations, for both NCs, removing the –R group is considered to be more feasible than losing the –SR group, and the exposed S site is likely to be the active center for CO_2_RR. In contrast, if –SR is removed, the exposed Au site is more selective for HER than for CO_2_RR. Furthermore, for Au_19_Cd_2_ NCs, the S active site (Cd–S–Au staple motif) will undergo a dynamic regeneration process during CO formation and desorption, contributing to the different *CO binding mode and leading to the decreased energy for CO formation (Fig. 6b). At the same time, Sun *et al.* investigated the CO_2_RR activities of Au_25_(SR)_18_, Au_24_Cd_1_(SR)_18_, Au_19_Cd_4_(SR)_18_, and Au_38_Cd_4_(SR)_30_, and found that Cd doping can selectively control the cleavage of the Au–S or C–S bond.^150^ They reached a similar conclusion: that is, the exposed open S site obtained by cleaving the C–S bond is readily bound to CO_2_ and favourable for CO_2_RR, while breakage of the Au–S bond leads to the exposure of the metal site, which is preferable for HER. Recognition of the real catalytic site in the two studies perfectly reflects the advantages of atomically precise NCs in revealing the catalytic mechanism and exploring the relationship between structure and activity.

In a comparison of CO_2_RR performance between Pd_1_Au_24_(SR)_18_ and Au_25_(SR)_18_ NCs (R = –CH_2_CH_2_Ph), Li *et al.* found that Pd_1_Au_24_ can greatly improve the CO_2_RR selectivity, with ∼100% FE_CO_, especially at high potential (up to −1.2 V), while the FE_CO_ of Au_25_ NCs starts to decrease at −0.9 V.^151^ Theoretical calculations demonstrate that the surface S atom is the active site, and Pd_1_Au_24_ can increase the thermodynamic barrier for ligand removal and retain a larger population of S active sites compared to Au_25_, which contributes to the improved CO_2_RR selectivity in the extended potential range. As Pd doping can effectively improve the activity of Au NCs in CO_2_RR, it is natural to think of two-element doping to form trimetallic Au-based NCs. Zhuang *et al.* utilized the AGR method to synthesize Au_3_AgPd_6_(TBBT)_12_ NCs by introducing a kernel Ag single atom on the basis of Au_4_Pd_6_(TBBT)_12_.^152^ The trimetallic Au_3_AgPd_6_ NCs show higher CO_2_RR activity and selectivity than Au_4_Pd_6_ with a similar structure (Fig. 6c). The introduction of Ag causes a significant change in electronic structure, decreases the d-center, weakens *CO adsorption, and promotes the release of CO, thereby improving the CO_2_RR activity. Meanwhile, the difficulty of *H desorption is increased, suppressing HER and enhancing CO_2_RR selectivity. Pd doping can not only enhance the activity of CO_2_RR but also shows the ability to change product type. Cai *et al.* prepared Au-based NCs (Au_9_ and Au_8_Pd_1_) confined in a layer of montmorillonite to form NC-based heterogenous catalysts.^153^ Pd doping can reduce the propensity for structural variation induced by the change in coordination number of the surface Au site during the reaction, bringing about multiple benefits to the catalysis. In a traditional fixed-bed reactor, Au_8_Pd_1_ exhibits unique catalytic performance in the CO_2_ hydrogenation reaction, changing the product type from methane to ethane, and improving the catalytic stability as well (Fig. 6d).

**Fig. 7:**
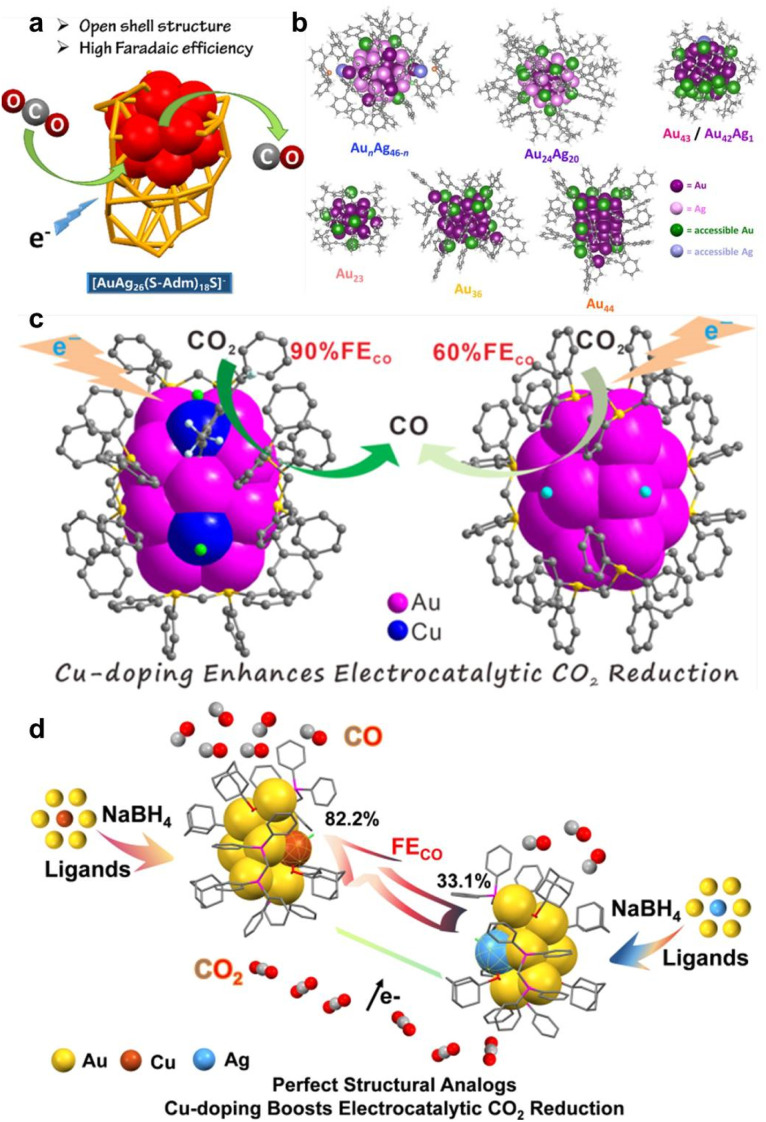
(a) Schematic diagram of open shell structure of AuAg_26_(SAdm)_18_S^−^ for enhanced CO_2_RR activity. Reproduced from ref. 155 with permission from American Chemical Society, copyright 2021. (b) The metal sites accessible to CO_2_ on the surface of different Au/Ag NCs. Reproduced from ref. 156 with permission from Royal Chemical Society, copyright 2023. (c) Schematic diagram of Au_15_Cu_4_-enhanced CO_2_RR activity compared to Au_18_ NCs. Reproduced from ref. 158 with permission from American Chemical Society, copyright 2023. (d) Comparison of CO_2_RR activity of structural analogs of Au_8_Cu_1_ and Au_8_Ag_1_ NCs. Reproduced from ref. 160 with permission from Wiley-VCH, copyright 2024.

Many reports have also shown that AuAg alloy NCs are a highly efficient electrocatalyst for CO_2_RR. Seong *et al.* developed an active-site transplantation strategy to synthesize core−shell AuAg_12_@Au_12_(SEtPh)_18_ NCs (SEtPh = 2-phenylethanethiolate) by replacing the Ag_12_(SR)_18_ shell of Ag_25_(SR)_18_ NCs with an Au_12_(SR)_18_ shell.^154^ Compared to Ag_25_(SR)_18_ NCs, the active-site-engineered AuAg_12_@Au_12_(SEtPh)_18_ NCs show significantly improved CO_2_RR activity with a 200 mA cm^−2^ industry current density and a 2.1 V full-cell potential in a zero-gap CO_2_-to-CO electrolyzer. Lin *et al.* prepared AuAg_26_(SAdm)_18_S^−^ (HSAdm = 1-adamantanethiolate) NCs composed of an AuAg_12_ icosahedron kernel and an Ag_14_(SR)_18_S open shell.^155^ Unlike Ag_25_(DMT)_18_^−^ (DMT = 2,4-dimethylbenzenethiol) and Au_21_(SAdm)_16_ with a closed shell, due to the open shell structure in AuAg_26_(SAdm)_18_S^−^, the partial Ag atoms of the AuAg_12_ kernel are not protected by thiols, resulting in the exposure of some facets of the kernel. Therefore, owing to the unique open shell structure and Au–Ag synergistic effect, AuAg_26_ exhibits lower *COOH formation free energy and displays increased CO_2_RR activity (98.4% FE_CO_ at −0.97 V), compared to Ag_25_ and Au_21_ NCs (Fig. 7a). Based on the superior CO_2_RR activity of AuAg alloy NCs, Li *et al.* further investigated the effect of the accessibility of metal sites on the CO_2_RR performance by systematically studying a series of alkynyl-protected AuAg NCs (Au_*n*_Ag_46−*n*_(C≡CR)_24_Cl_4_(PPh_3_)_2_, Au_24_Ag_20_(C≡CR)_24_Cl_2_, and Au_43_(C≡CR)_20_/Au_42_Ag_1_(C≡CR)_20_) of similar size and structure but different surface ligand coverage.^156^ Among these NCs, Au_43_(C≡CR)_20_ and Au_42_Ag_1_(C≡CR)_20_ with the highest number of accessible metal sites exhibit the highest FE_CO_ (Fig. 7b). Xu *et al.* utilized two AuAg alloy NCs (Au_24_Ag_20_ and Au_43_Ag_38_) to explore how the hierarchical assembly influences CO_2_RR activity.^157^ There are some correlations and differences between the two NCs, such as Au_43_Ag_38_ maintaining the kernel framework from the parent Au_24_Ag_20_ and it can be regarded as a dimeric form of Au_24_Ag_20_ monomeric NCs; Au_24_Ag_20_ is racemic, while Au_43_Ag_38_ is mesomeric; Au_24_Ag_20_ exhibits superatomic electronic configurations, while Au_43_Ag_38_ has molecule-like characteristics. Possibly due to the different atomic packing structures (individual-core *vs.* dual-core) and surface motif arrangement (parallel *vs.* crossed), the Au_24_Ag_20_ monomers show better CO_2_RR activity than Au_43_Ag_38_ dimers.

**Fig. 8:**
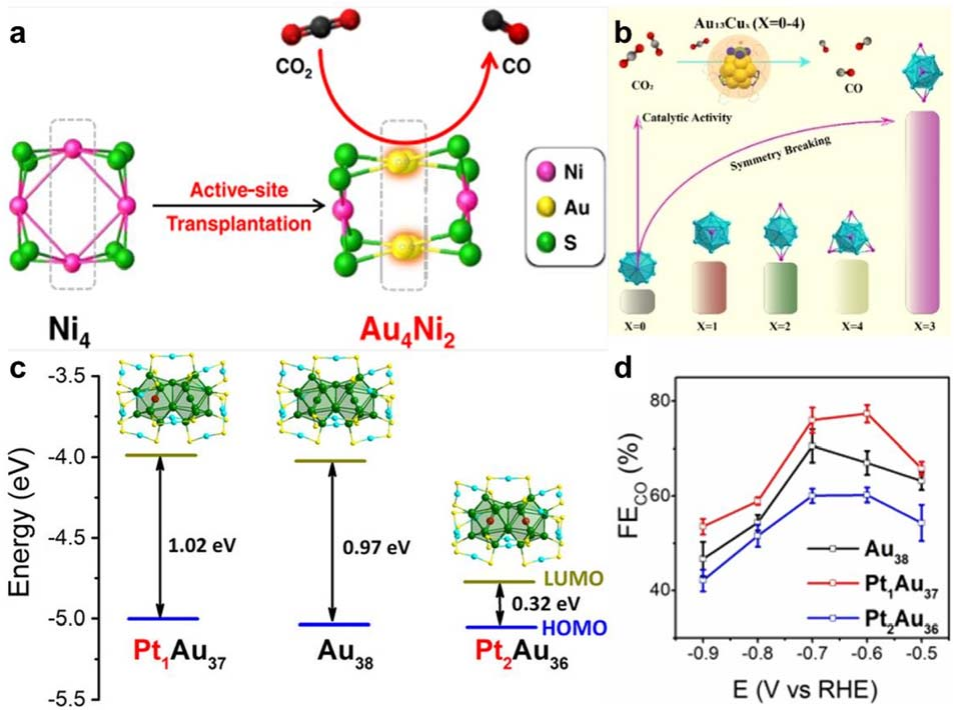
(a) Schematic diagram of active site transplantation from Ni_4_ to Au_4_Ni_2_ NCs for enhanced CO_2_RR. Reproduced from ref. 161 with permission from American Chemical Society, copyright 2023. (b) Schematic diagram of the different degrees of symmetry breaking in Au_13_Cu_*x*_ (*x* = 0–4) NCs for different CO_2_RR performances. Reproduced from ref. 166 with permission from Wiley-VCH, copyright 2024. (c) Electronic structure energy level and (d) comparison of CO_2_RR activity of Au_38_, Pt_1_Au_37_, and Pt_2_Au_36_. Reproduced from ref. 167 with permission from Wiley-VCH, copyright 2022.

Cu is considered as a special element for CO_2_RR due to the suitable adsorption energy for *CO and *CH_*X*_O intermediates; therefore Cu-doped Au NCs have received a lot of attention. Deng *et al.* synthesized [Au_15_Cu_4_(DPPM)_6_Cl_4_(C≡CR)_1_]^2+^ NCs (DPPM = bis(diphenylphosphino)methane; HC≡CR = 3,5-bis-(trifluoromethyl)phenylacetylene) *via* a one-pot method.^158^ Compared to the monometallic structural analogue, [Au_18_(DPPM)_6_Br_4_]^2+^, Au_15_Cu_4_ shows a dramatic enhancement in CO_2_RR activity with a high FE_CO_ of >90% and an up to industrial CO partial current density of −413 mA cm^−2^ at −3.75 V in a membrane electrode assembly (MEA) cell (Fig. 7c). The exposed Au–Cu dual sites synergistically modulate the d-state shift, contributing to the enhanced catalytic activity. Ding *et al.* also explored the Au–Cu synergistic effect on CO_2_RR activity.^159^ They found that Au_1_Cu_24_ NCs are preferable for the CO_2_RR, while Cu_25_ NCs tends to progress HER, because AuCu synergy can effectively suppress HER due to the contracted electronic distribution. Based on the single Cl-terminated coordination strategy, Su *et al.* obtained a pair of structural analogs, [Au_8_Ag_1_(SAdm)_4_(Dppm)_3_Cl]^2+^ and [Au_8_Cu_1_(SAdm)_4_(Dppm)_3_Cl]^2+^.^160^ Due to the same overall structure and metal doping number and position, the metal doping type effect can be decoupled from various entangled influencing factors to elucidate metal synergies solely in terms of atomic differences. Au_8_Cu_1_ shows higher FE_CO_ than Au_8_Ag_1_ due to the generation of thermodynamically more stable *COOH and lower *CO formation energy (Fig. 7d). The rare structural analogs help us clearly reveal the impact of single-metal change on the catalytic performance. Doping a foreign metal atom to form Au-based alloy NCs can not only regulate the electronic and geometric structure to enhance catalytic activity but also minimize Au usage and reduce the catalyst cost when doping with non-precious metal atoms. Seong *et al.* developed a highly-efficient and economic CO_2_RR catalyst with high mass activity, Au_4_Ni_2_ NCs, by the transplantation of Au active sites into non-precise Ni_4_ NCs (Fig. 8a).^161^ In Au_4_Ni_2_ NCs, the Au atoms are the active sites, the Ni atoms mainly play the role of a cost reducer, and thereby the utilization efficiency of the Au atoms is improved. The TOF and mass activity of Au_4_Ni_2_ NCs for CO_2_-to-CO are 206 mol_CO_ mol_NC_ s^−1^ and 25228 A/g_Au_, respectively.

For CO_2_RR, the activation of CO_2_ is critical because it is the first step in CO_2_ conversion. CO_2_ is a linear molecule with high stability, resulting in difficulty in its activation.^162^ Constructing asymmetric charge distribution sites is an effective way to activate CO_2_,^163,164^ and our previous review introduced several strategies of symmetry-breaking sites in metal NCs for enhancing CO_2_RR activity, including a heteroatom doping induced strategy.^165^ For example, Tan *et al.* synthesized a series of Au_13_Cu_*x*_ alloy NCs (*x* = 0–4) with different degrees of symmetry-breaking on the crystal structure by manipulating Cu atom doping (Fig. 8b).^166^ Au_13_Cu_1_, Au_13_Cu_2_, and Au_13_Cu_4_ have *C*_3V_ symmetry, *C*_3h_ symmetry, and *T*_d_ symmetry, respectively, while Au_13_Cu_3_ exhibits an asymmetric surface structure with 2 three-coordinate Cu_1_S_3_ and 1 two-coordinate Cu_1_S_2_ motifs. With the highest asymmetry degree of Au_13_Cu_3_, the unsaturated Cu_1_S_2_ site on the surface more readily coordinates with CO_2_, promoting CO_2_ adsorption and CO_2_RR activity. As a result, Au_13_Cu_3_ shows the highest current density of 70 mA cm^−2^ within the Au_13_Cu_*x*_ family of NCs (Au_13_Cu_*x*_ (*x* = 1, 2, 4): 40 mA cm^−2^, and Au_13_ : 15 mA cm^−2^). In addition, Liu *et al.* obtained identical geometric structural Pt_1_Au_37_(SR)_24_ and Pt_2_Au_36_(SR)_24_ (SR = 4-*tert*-butylbenzyl mercaptan) by the controllable asymmetrical and symmetrical doping of Pt atoms into parent Au_38_(SR)_24_ NCs.^167^ The structure of Pt_1_Au_37_ is that one Pt atom asymmetrically dopes into one of the two cores of Au_38_, and relative to Au_38_, Pt_1_Au_37_ displays higher HOMO energy with the loss of one valence electron. While the two Pt atoms in Pt_2_Au_36_ are symmetrically located in the two cores of Au_38_, and Pt_2_Au_36_ exhibits a narrowed HOMO–LUMO gap and loses two valence electrons, compared to Au_38_ (Fig. 8c). The difference in doping mode causes the modulation of electronic structure; thereby, Pt_1_Au_37_ shows high electron-spin-induced CO_2_RR activity, followed by Au_38_ and Pt_2_Au_36_ (Fig. 8d). Besides, Wang *et al.* utilized an Fe single atom (SA) site to modify Au_8_ NCs and integrate SA and NC into one system.^168^ Due to the introduction of Fe SA, asymmetric charge distributed active sites are constructed and the key intermediate *COOH adsorption energy is optimized to benefit an improvement in CO_2_RR activity, leading to ∼18.07-fold amplification in the FE of CO_2_-to-CO compared to isolated Au_8_ NCs.

### 3.6 Au-based bimetallic NCs in the synthetic ammonia reaction

Ammonia is an important industrial raw material that plays an important role in the manufacture of fertilizers, dyes, and pharmaceuticals. In addition, due to its large hydrogen capacity, high energy density, and ease of transportation, ammonia is considered a potential carbon-free fuel.^169–171^ The traditional Haber–Bosch process enables the large-scale industrial production of ammonia, but its production conditions (400–550 °C, 15–30 MPa) are harsh and are associated with high energy consumption and CO_2_ emissions.^172–174^ Using sustainable electric energy, solar energy and other new energy sources as the driving force, abundant N_2_ in the atmosphere or environmentally harmful but highly active nitrogen-containing species (such as NO, NO_2_^−^/NO_3_^−^) are reduced to synthesize ammonia under conditions of ambient temperature and pressure, which is considered to have great application potential.^20,175^

**Fig. 9:**
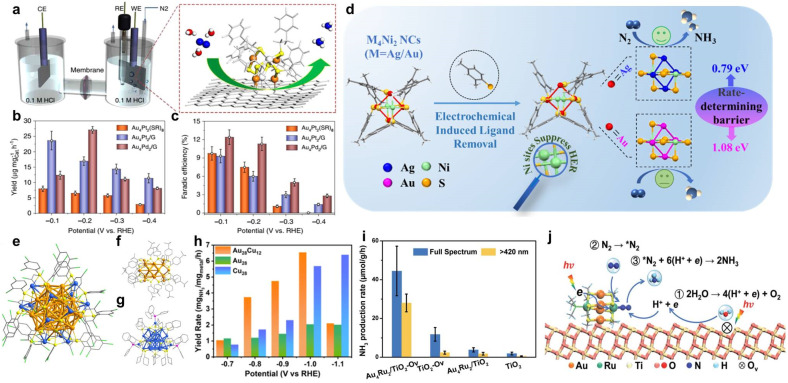
(a) Schematic diagram of Au_4_M_2_/G (M = Pt, Pd) for NRR. (b) NH_3_ yield rate and (c) FE of NH_3_ comparison of Au_4_Pt_2_ NC and Au_4_M_2_/G (M = Pt, Pd). Reproduced from ref. 176 with permission from Springer Nature, copyright 2020. (d) Schematic diagram of regulation of intrinsic activity of M_4_Ni_2_ (M = Au, Ag) NCs for NRR. Reproduced from ref. 177 with permission from Wiley-VCH, copyright 2022. (e) Structure of Au_28_Cu_12_, (f) Au_28_, and (g) Cu_28_ NCs. (h) NH_3_ yield rate comparison of Au_28_Cu_12_, Au_28_, and Cu_28_ NCs. Reproduced from ref. 181 with permission from Royal Chemical Society, copyright 2024. (i) NH_3_ yield rate of Au_4_Ru_2_/TiO_2_–O_v_, TiO_2_–O_v_, Au_4_Ru_2_/TiO_2_, and TiO_2_ under different sources of light irradiation. (j) Proposed mechanism for photocatalytic NRR on Au_4_Ru_2_/TiO_2_–O_v_. Reproduced from ref. 182 with permission from Royal Chemical Society, copyright 2020.

Yao *et al.* firstly synthesized Pt/Pd-doped Au-based NCs (Au_4_M_2_(SR)_8_, M = Pd/Pt) with a precise and controllable size distribution and metal doping and then removed partial ligands by thermal treatment to anchor it onto defective graphene to create a robust supported NC-based catalyst (Fig. 9a).^176^ DFT calculations indicate that heteroatom doping plays an essential role in N_2_ activation *via* enhanced electron back donation to N_2_ antibonding π*-orbitals. By precise metal doping, Au_4_Pd_2_ shows higher FE_NH3_ and NH_3_ yield at −0.2 V than Au_4_Pt_2_ in the electrocatalytic N_2_ reduction reaction (NRR) (Fig. 9b and c). From the perspective of tuning intrinsic catalytic activity of NCs, Han *et al.* introduced non-precise metal Ni with the ability to suppress HER (the competitive reaction to NRR) to prepare M_4_Ni_2_ (M = Au/Ag) NCs.^177^ Compared to monometallic NCs (Au_6_, Ag_6_, and Ni_6_), bimetallic M_4_Ni_2_ (M = Au/Ag) shows better NRR performance. On the one hand, the superior NRR activity is attributed to the effective inhibition of HER by the introduction of Ni. On the other hand, partial ligand detachment during the electrochemical process provides exposed active sites to access more N_2_ reactant and induces reconstruction of the electronic structure, benefiting the NRR (Fig. 9d).

It has been reported that Cu is considered to be a promising candidate for electrocatalytic NO_3_^−^ reduction (NO_3_RR) to NH_3_.^178^ However, Cu is prone to surface poisoning due to spontaneous oxidative dissolution as well as easy adsorption of other species in the electrolyte.^179,180^ Tang *et al.* doped Cu atoms on the surface of Au_28_ NCs to form stable AuCu alloy NCs (Au_28_Cu_12_(SR)_24_(PPh_4_)_4_ (SR = 2,4-dichlorothiophenol).^181^ Due to the synergistic effect between Au and Cu, the geometric and electronic structure of Au_28_Cu_12_ are tailored to be suitable for NO_3_RR. Compared to monometallic Au_28_(TBBT)_20_ (TBBT = 4-*tert*-butylbenzenethiol) and Cu_28_(CHT)_18_(PPh_3_)_3_ (CHT = cyclohexanethiol), bimetallic Au_28_Cu_12_ shows higher NH_3_ production activity and stability (Fig. 9e–h).

Sun *et al.* first screened the feasibility of light-driven NRR by a series of Au_n_ NCs (*n* = 8, 9, 11, 18, 23, 24, 25, 28, 36, 44).^182^ Due to the physical adsorption of N_2_ on the surface of Au_*n*_ (the distance between N_2_ and Au_*n*_ is longer than 3.2 Å), it is impossible for N_2_ to coordinate to Au_*n*_ for activation. Considering Ru is a recognized NRR candidate, Ru-doped Au NCs (Au_4_Ru_2_(PPh_3_)_2_(SC_2_H_4_Ph)_8_) are synthesized and then loaded onto the oxygen vacancies of TiO_2_ to form an NC-based heterogeneous photocatalyst (Au_4_Ru_2_/TiO_2_–O_v_) (Fig. 9i). Under light irradiation, both Au_4_Ru_2_ NCs and TiO_2_ can directly generate e–h pairs and photoinduced electron transfer from TiO_2_ to Au_4_Ru_2_ NCs. The introduction of Ru atoms can effectively promote electron injection to adsorbed N_2_ and then realize N_2_ activation (Fig. 9j). The supported bimetallic Au_4_Ru_2_ NCs displays better photocatalytic NRR activity than homogold Au_*n*_ NCs (Fig. 9i). This work not only indicates the ability for light harvesting and photogenerating e–h pairs over NCs with non-metallic or excitonic behavior, but also demonstrates a cooperative effect based on heteroatom doping.

## 4. Summary and outlook

Metal NCs not only act as a bridge connecting metal NPs and SACs in terms of their size aspect, it also compensates for their shortcomings in catalysis as a unique type of nanomaterial. First of all, from the perspective of electronic structure, metal NCs have discrete electronic energy levels unlike metal NPs and SACs, and therefore, they show distinctive performance in catalysis. Secondly, compared to metal NPs, the size of the metal NCs is smaller, their atomic utilization is higher, and the number of surface unsaturated coordinated active sites is richer. In addition, the molecule-like high purity and homogeneity in size, composition, structure, and surface environment of atomically precise metal NCs provides the opportunity to reveal the catalytic mechanism and guide catalyst design at the atomic level, which is impossible for metal NPs to achieve. Compared to SACs, metal NCs can effectively exert a synergistic effect between metal atoms, adjusting the composition, geometric configuration and electronic structure of the catalyst, to realize “1 + 1 > 2” enhancement of catalytic activity, selectivity and stability.

In the metal NC family, Au-based NCs not only exhibit the advantages of simple synthesis, high yield, and easy functionalization but also possess the characteristics of high stability and solubility in various solvents, therefore attracting lots of attention from catalysis researchers. Unlike previous reviews on alloy NCs and NC catalysis (either focusing on the controllable synthesis of NCs or involving general catalytic reactions), this perspective aims at the unsatisfactory activity and dilemma about the unclear reaction mechanism facing the energy-related small-molecule catalytic reaction at the present stage, mainly summarizing the synergistic effect on an enhancement in catalytic performance in atomically precise Au-based bimetallic NCs. From the above summary and discussion, great efforts have been made in this field, and gratifying developments and advances have been achieved. Finally, in this section, some of our own perspectives and insights are put forward, to promote further development, innovation and breakthroughs in this field in the future.

(1) The synthesis range of doped metal NCs needs to be further expanded. It is not limited to bimetallic NCs. At present, there have been reports on trimetallic alloy NCs. We can learn from ideas about the preparation of high-entropy alloy NPs,^183,184^ to try to synthesize polymetallic alloy NCs, and perhaps some novel and unexpected phenomena or properties will appear in the subnanometer region. In addition, as far as synthesis is concerned, the rapid development of society no longer allows us to continue to blindly use the “trial and error” approach, which is too inefficient. We can use the advanced scientific and technological achievements of computer science, allowing chemistry, catalysis, computer technology and other interdisciplinary subjects to cross-fertilise and integrate, use artificial intelligence to predict the kind of doping, the amount of doping, and the position of doping for the best catalytic performance, which can greatly improve preparation efficiency.^185,186^ Additionally, they can be combined with a high-throughput NC synthesis technique to quickly prepare multiple types of NCs at one time in order to quickly screen their catalytic properties.^187^

(2) Considering future practical application scenarios, the cost-effectiveness of catalysts is an important consideration. It is necessary to develop non-noble metal clusters, such as transition metal clusters (iron-based, cobalt-based, nickel-based, copper-based clusters, *etc.*). However, due to their inherent instability (easily reacting with oxygen and water), the synthesis conditions are relatively harsh, and the purity and yield of the obtained product are unsatisfactory. Therefore, it is necessary to choose more suitable protective ligands to stabilize the transition metal core. In addition, synthesis can also be considered in an anhydrous and oxygen-free environment in a glove box. The large-scale preparation of cluster-based catalysts also needs to be given attention. Currently, chemical synthesis methods are relatively mature, and there are many types of clusters that can be prepared by this method, and high-purity and high-yield products can be obtained through precise control over chemical reaction conditions. However, the synthesis of cluster-based catalysts is still in the laboratory stage, and there are only a few reports in the literature where the yield of one-time synthesis reaches the gram level.^188–191^ There is still a long way to go to achieve industrial-scale preparation. Furthermore, for the recycling and recovery of clusters after catalytic reaction, one strategy with the most potential and promise is the construction of cluster-based composites. Due to the small size of the clusters, a support is required to load them, while avoiding aggregation or mass loss after the reaction. Porous materials are good candidates due to their abundant and adjustable pores and channels. If the surface of the material could be modified with various functional groups, it would be even better because it could solidly connect with clusters, and various types of surface groups may also bring about unexpected improvements in catalytic performance.

(3) For bimetallic NCs, and even for polymetallic NCs that may be used in the future, the enhancement in their catalytic performance is mainly attributed to the synergistic effect between metal atoms, but this statement is very general. Specifically, it is not particularly clear how the components affect each other or regulate each other, and how they play a synergistic role. Do the different metal atoms regulate each other's electronic structure, jointly affect the adsorption, activation and desorption behaviour of reaction molecules/intermediates/product molecules, and then realize synergistic catalysis? or is it tandem/cascade catalysis? for example, in CO_2_RR, in an Au–Cu alloy, the Au site usually plays the role of converting CO_2_ into CO, and then CO reduction and coupling continue to occur at the Cu site, achieving efficient tandem/cascade catalysis.^192^ This is a scientific question worthy of detailed discussion and careful investigation.

(4) An NC is a complex system involving an inner core of metal atoms and outer organic ligands, each of which plays a role in catalysis. In some catalytic cases, it has been found that it is not only the surface atoms that participate in the catalytic reaction; even the metal atoms at the center of the metal core also contribute to catalysis.^153,167^ Additionally, in cases of the Au–Cd NCs catalysis of CO_2_RR, the real catalytically active center (the exposed Au site with –SR detachment, or the exposed S site with –R removal) has been investigated in detail.^149,150^ All these inspire us to make more careful explorations in the future to determine the real active sites. In addition, for an expansion of types of energy catalysis, especially those involving multiple reactants and steps, it will be meaningful and useful to clarify the real role each component plays in catalysis to guide catalyst design in the future.

(5) Although most current energy catalytic reactions are still laboratory-level, the future will certainly tend toward industrialization, and there are already some reports about HER, ORR, CO_2_RR, *etc.* at industrial current density at this stage.^193–195^ As mentioned above, using NCs alone still faces the problem of easy aggregation and declining activity. Therefore, in industrial catalysis, functional supports are needed to load NCs to strengthen their stability and increase service lifetime. Therefore, it is necessary to clarify the interface and interaction between NCs and supports, so as to purposefully select appropriate supports and form supported NC-based catalysts with high activity for target reactions.

Overall, atomically precise bimetallic NCs have unique advantages in energy-related catalysis not only reflecting their extraordinary activity but also providing the opportunity to build structure–activity correlation; therefore, it deserves much more effort for further in-depth exploration and wide application, and the insights and general rules obtained from it that can be extended to other alloy materials and more types of catalytic reaction in the future.

## Data availability

All data in this perspective were cited from other references.

## Author contributions

Y. X. Du and M. Z. Zhu conceived the topic and structure of the article. Y. Fang and P. Wang conducted the literature research and designed the figures and tables. All authors were responsible for reviewing, editing and developing the perspective.

## Conflicts of interest

The authors have no competing interests to declare.

## Appendix

**Table 1:** Summary of representative Au-based bimetallic NCs

Series NC_S_ name	NC_S_ formula	Synthesis method	Structural characteristic			Reference
			Doping type	Doping number	Doping position	
M_1_Au_3_ series NCs	[(Audppy)_3_AgO](BF_4_)_2_	Metal-exchange strategy	Ag	1	Kernel surface doping	196
M_2_Au_4_ series NCs	Au_4_Ni_2_(PPh_2_)_2_S_2_(PCP)_2_	Direct co-reduction strategy	Ni	2	Kernel surface doping	197
	Au_4_Pd_2_(SR)_8_, SR = SC_2_H_4_Ph	Direct co-reduction strategy	Pd	2	Kernel surface doping	105
	Pt_2_Au_4_L_8_, L = C_21_H_28_O_2_	Direct co-reduction strategy	Pt	2	Kernel surface doping	198
M_4_Au_4_ series NCs	[Au_4_Cu_4_(π-CH≡Cp-C_6_H_4_R)], R = H, Cl, CH_3_	Direct co-reduction strategy	Cu	4	Kernel surface doping	199
	Au_4_Cd_4_(2, 4-DMBT)_12_	Cation-assisted strategy	Cd	4	Kernel surface doping	200
	[Au_4_Cu_4_(DPPM)_2_(S-Adm)_5_]^+^Br^−^	Direct co-reduction strategy	Cu	4	Kernel surface doping	201
M_2_Au_6_ series NCs	[Au_6_Ag_2_(C)(L_2_)_6_](BF_4_)_4_, L2 = 2-(diphenylphosphino)-5-pyridinecarboxaldehyde	Direct co-reduction strategy	Ag	2	Kernel surface doping	202
	[Au_6_Ag_2_C(dppy)_6_](BF_4_)_4_	Direct co-reduction strategy	Ag	2	Kernel surface doping	203
M_6_Au_2_ series NCs	Au_2_Cu_6_(PPh_2_Py)_2_(S-Adm)_6_, Au_2_Cu_6_(PPh_2_Py)_2_(TBM)_6_	Direct co-reduction strategy	Cu	6	Kernel surface doping	204
	Au_2_Cu_6_(S-Adm)_6_(P(Ph-OMe)_3_)_2_, Au_2_Cu_6_(S-Adm)_6_(PPh_3_)_2_, Au_2_Cu_6_(S-Adm)_6_(P(Ph-F)_3_)_2_	Direct co-reduction strategy	Cu	6	Kernel surface doping	205
(M–Au)_9_ series NCs	[PdAu_8_(PPh_3_)_8_]^2+^	Direct co-reduction strategy	Pd	1	Center doping	206
	Au_4_Ag_5_(dppm)_2_(S-Adm)_6_-(BPh_4_)	Direct co-reduction strategy	Ag	5	Kernel surface doping	207
(M–Au)_10_ series NCs	Au_4_Pd_6_(TBBT)_12_	Direct co-reduction strategy	Pd	6	Kernel surface doping	152
	[PdAu_9_H(PPh_3_)_8_Cl]^+^	Direct co-reduction strategy	Pd	1	Center doping	208
(M–Au)_12_ series NCs	[Au_10_Ag_2_(2-py-C=C)_3_(dppy)_6_](BF_4_)_5_	Direct co-reduction strategy	Ag	2	Kernel surface doping	209
	[Au_11_Cu_1_(PPh_3_)_7_(SPy)_3_]^+^	Direct co-reduction strategy	Cu	1	Kernel surface doping	210
	Au_6_Cu_6_MBT_12_, Au_6_Cu_6_PET_12_	Metal-exchange strategy	Cu	6	Kernel surface doping	211
	AuCu_11_[S_2_P(OiPr)_2_]_6_(C≡CPh)_3_Cl	Intercluster reaction strategy	Cu	11	Kernel surface doping	212
(M–Au)_13_ series NCs	[AuCu_12_(SR)_6_(C≡CPh)_4_]^+^, SR = S_2_P(C_2_H_4_Ph)_2_	Direct co-reduction strategy	Cu	12	Kernel doping	213
(M–Au)15 series NCs	[Au_7_Ag_8_(^*t*^BuC≡C)_12_]^+^	Direct co-reduction strategy	Ag	8	Kernel surface doping	214
(M–Au)_16_ series NCs	[Au_7_Ag_9_(dppf)_3_(CF_3_CO_2_)_7_BF_4_]_n_	Direct co-reduction strategy	Ag	9	Kernel surface doping, Motif doping	215
Au_13_Cux series NCs	[Au_13_Cu_4_(PPh_2_Py)_4_(SC_6_H_4_-*tert*-C_4_H_9_)_8_]^+^	Intercluster reaction strategy	Cu	4	Motif doping	216
	Au_13_Cu_4_(PPh_2_Py)_3_(SePh)_9_	Direct co-reduction strategy	Cu	4	Motif doping	217
	[Au_13_Cu_2_(DPPP)_3_(SPy)_6_]^+^	Direct co-reduction strategy	Cu	2	Motif doping	218
	[Au_13_Cu_2_(PPh_3_)_6_(SPy)_6_]^+^	Intercluster reaction strategy	Cu	2	Motif doping	216
(M–Au)_17_ series NCs	Au_16_Ag_1_(S-Adm)_13_	Direct co-reduction strategy	Ag	1	Center doping	219
	Au_4_Ag_13_(DPPM)_3_(SR)_9_, SR = C_8_H_10_S	Direct co-reduction strategy	Ag	13	Kernel surface doping, Motif doping	220
	Au_*x*_Ag_17−x_N_3_(TBBT)_12_, N = a counter cation, *x* = 0, 1	Direct co-reduction strategy	Ag	16, 17	Kernel doping	221
(M–Au)_18_ series NCs	Au_15_Ag_3_(SR)_14_ SR=SC_6_H_11_	Intercluster reaction strategy	Ag	3	Kernel surface doping	222
	[Ag_*x*_Au_18−x_(Dppm)_6_Br_4_](BPh_4_)_2_ (*x* = 1, 2)	Metal-exchange strategy	Ag	1, 2	Kernel surface doping	223
(M–Au)_19_ series NCs	[Au_7_Cu_12_(dppy)_6_(TBBT)_6_Br_4_]^3+^	Direct co-reduction strategy	Cu	12	Kernel surface doping	224
(M–Au)_21_ series NCs	[Au_19_Cd_2_(SR)_16_]^–^, SR = C_6_H_11_S	Direct co-reduction strategy	Cd	2	Motif doping	225
	[Au_9_Ag_12_(SR)_4_(dppm)_6_X_6_]^3+^, SR = S-Adm/S-^*t*^Bu, *X* = Cl/Br	Direct co-reduction strategy	Ag	12	Kernel surface doping, Motif doping	226
	Au_20_Ag_1_(S-Adm)_15_	Direct co-reduction strategy	Ag	1	All	227
	Au_20_Ag_1_(SR)_15_, Au_21–*x*_Ag_*x*_(SR)_15_ (*x* = 4–8), Au_21–*x*_Cu_*x*_(SR)_15_ (*x* = 0–5), SR = SC_4_H_10_O	Direct co-reduction strategy	Ag, Cu	0–8	Kernel surface doping/Motif doping	228
(M–Au)_23_ series NCs	Au_23-*x*_Cu_*x*_(SR)_16_, SR = SC_6_H_12_O	Direct co-reduction strategy	Cd	2	Motif doping	225
M_1_Au_24_ series NCs	[Au_24_Pd(PPh_3_)_10_(SR)_5_Cl_2_], SR = SC_2_H_4_Ph	Direct co-reduction strategy	Pd	1	Center doping	229
	Au_24_M_1_(SR)_18_, SR = SC_2_Ph/PET	Direct co-reduction strategy	Hg, Cd, Pt, Pd	1	Kernel surface doping/Center doping	230–232
M_24_Au_1_ series NCs	[Ag_24_Au(SR)_18_]^–^ PPh_4_^+^, SR = SPhMe_2_	Direct co-reduction strategy	Ag	24	Kernel surface doping, Motif doping	233
	[Au_1_Ag_24_(Dppm)_3_(SR)_17_]^2+^, SR = C_6_H_12_S	Direct co-reduction strategy	Ag	24	Kernel surface doping, Motif doping	234
	AuCu_24_H_22_(PPh_3_)_12_, AuCu_24_H_22_((*p*-FPh)_3_P)_12_	Direct co-reduction strategy	Cu	24	Kernel surface doping	189
(M–Au)_25_ series NCs	[Cu_13_Au_12_(PPh_3_)_10_(SR)_5_Cl_2_]^2+^, SR = PhC_2_H_4_S	Direct co-reduction strategy	Cu	13	Kernel surface doping	235
	Au_22_Ir_3_(PET)_18_	Intercluster reaction strategy	Ir	3	Kernel doping	236
	Pd_2_Au_23_(PPh_3_)_10_Br_7_	Direct co-reduction strategy	Pd	2	Center doping	237
(M–Au)_27_ series NCs	[Au_4_Ag_23_(C≡ C^*t*^Bu)_10_(dppf)_4_Cl_7_](PF_6_)_2_	Direct co-reduction strategy	Ag	23	Kernel surface doping, Motif doping	238
	Au_25_Ag_2_(SR)_18_, SR = SC_2_H_4_Ph	Direct co-reduction strategy	Ag	2	Motif doping	239
(M–Au)_29_ series NCs	Ag_29−*x*_Au_*x*_(SR)_12_(pph)_4_, SR = S_2_C_6_H_6_ (*x* = 1–5)	Direct co-reduction strategy	Ag	24–28	Kernel surface doping, Motif doping	240
	[Au_5_Ag_24_(C=CC_6_H_4_-*p*-^*t*^Bu)_16_(dppf)_4_Cl_4_](PF_6_)_3_	Direct co-reduction strategy	Ag	24	Kernel surface doping, Motif doping	238
(M–Au)_30_ series NCs	Au_24_Cu_6_(SPh^*t*^Bu)_22_	Intercluster reaction strategy	Cu	6	Motif doping	241
(M–Au)_34_ series NCs	[Ag_33_AuS_2_(SR)_18_(CF_3_COO)_9_(DMF)_6_]	Direct co-reduction strategy	Ag	33	Kernel surface doping, Motif doping	242
	[AuAg_33_(BTCA)_3_(C≡Cbu^*t*^)_9_(tfa)_4_(CH_3_OH)_3_]SbF_6_	Direct co-reduction strategy	Ag	33	Kernel surface doping, Motif doping	243
(M–Au)_38_ series NCs	Au_38−x_Cu_*x*_(2,4-(CH_3_)_2_C_6_H_3_S)_24_ (*x* = 0–6)	Intercluster reaction strategy	Cu	0–6	Motif doping	244
M_2_Au_36_ series NCs	M_2_Au_36_(SR)_24_, SR = SC_2_H_4_Ph/SC_6_H_13_	Direct co-reduction strategy	Pd, Pt	2	Center doping	245–248
(M–Au)_40_ series NCs	AuAg_39_(TBBM)_21_(CH_3_COO)_11_	Intercluster reaction strategy	Ag	39	Kernel surface doping, Motif doping	249
(M–Au)_41_ series NCs	[Au_3_Ag_38_(SR)_24_X_5_]^2−^ (*X* = Cl or Br), SR = SCH_2_Ph	Direct co-reduction strategy	Ag	38	Kernel surface doping, Motif doping	250
(M–Au)_42_ series NCs	Au_38_Cd_4_(DMBT)_30_	Metal-exchange strategy	Cd	4	Kernel surface doping	251
(M–Au)_44_ series NCs	Au_12_@S_8_@Ag_32_(PS)_24_]^2+^	Direct co-reduction strategy	Ag	32	Kernel surface doping	252
	[Au_12+x_Cu_32_(SR_)30+x_]^4–^, SR = SPhCF_3_	Direct co-reduction strategy	Cu	32	Motif doping	253
	Au_24_Ag_20_(2-SPy)_4_(PhC≡C)_20_Cl_2_	Direct co-reduction strategy	Ag	6	Kernel surface doping	254
	Au_2_Ag_42_(S-Adm)_27_(BPh_4_)	Direct co-reduction strategy	Ag	42	Kernel surface doping, Motif doping	255
M_32_Au_12_ series NCs	[Au_12_Ag_32_(FTP)_30_]^4–^	Metal-exchange strategy	Ag	32	Kernel surface doping, Motif doping	256
	Au_12_Ag_32_(SR)_30_ SR = SPhF/SPhF_2_/SPhCF_3_	Direct co-reduction strategy	Ag	32	Kernel surface doping, Motif doping	190
(M–Au)_45_ series NCs	Au_9_Ag_36_(SR)_27_(PPh_3_)_6_, SR = SPhCl_2_	Direct co-reduction strategy	Ag	36	Kernel surface doping, Motif doping	257
(M–Au)_48_ series NCs	Au_26_Ag_22_(TBBT)_30_	Direct co-reduction strategy	Ag	22	Kernel surface doping, Motif doping	258
(M–Au)_49_ series NCs	[Au_19_Cu_30_(C=CR)_22_(Ph_3_P)_6_Cl_2_](NO_3_)_3_, RC=C=H_3_C_4_S-_3_—C≡CH/PhC≡CH	Direct co-reduction strategy	Cu	30	Kernel surface doping	259
(M–Au)_50_ series NCs	[Au_2_Ag_48_(S-^*t*^Bu)_20_-(Dppm)_6_Br_11_]Br(BPh_4_)_2_	Direct co-reduction strategy	Ag	48	Kernel surface doping, Motif doping	255
(M–Au)_62_ series NCs	Au_34_Ag_28_(PhC≡C)_34_	Direct co-reduction strategy	Ag	28	Kernel surface doping	260
(M–Au)_70_ series NCs	[Ag_46_Au_24_(S^*t*^Bu)_32_](BPh_4_)_2_	Direct co-reduction strategy	Ag	46	Kernel doping	261
(M–Au)_110_ series NCs	[Au_80_Ag_30_(C≡CPh)_42_Cl_9_]Cl	Direct co-reduction strategy	Ag	30	Kernel surface doping	262
	Au_57_Ag_53_(C≡CPh)_40_Br_12_	Direct co-reduction strategy	Ag	53	All	263
(M–Au)_124_ series NCs	[Au_52_Cu_72_(*p*-MBT)_55_]^+^Cl^−^, *p*-MBT = SPh-*p*-CH_3_	Direct co-reduction strategy	Cu	72	Kernel doping	264
(M–Au)_130_ series NCs	Au_130−*x*_Ag_*x*_(TBBT)_55_	Direct co-reduction strategy	Ag	98	All	265

**Table:** 

Ligand abbreviations	Formula
dppy	C_17_H_14_NP
pph	C_18_H_15_P
dppm	C_25_H_22_P_2_
S-Adm	C_10_H_16_S
2,4-DMBT	C_8_H_10_S
TBM	C_22_H_18_
py	C_5_H_5_N
TBBT	C_10_H_14_S
S-*t*Bu	C_4_H_10_S
dppf	C_34_H_28_FeP_2_
PET	C_8_H_10_S
CHM	C_6_H_12_S
SSR	C_6_H_6_S_2_
BTCA	C_8_H_10_O_8_
